# Outcome choice and definition in systematic reviews leads to few eligible studies included in meta-analyses: a case study

**DOI:** 10.1186/s12874-020-0898-2

**Published:** 2020-02-11

**Authors:** Ian J. Saldanha, Kristina B. Lindsley, Sarah Money, Hannah J. Kimmel, Bryant T. Smith, Kay Dickersin

**Affiliations:** 1 Center for Evidence Synthesis in Health, Department of Health Services, Policy, and Practice (Primary), Department of Epidemiology (Secondary), Brown University School of Public Health, 121 South Main Street, Box G-S121-8, Providence, RI 02903 USA; 2 Julius Center for Health Sciences and Primary Care, University Medical Center Utrecht, Utrecht University, Room Str. 6.127, Utrecht, GA 3508 Netherlands; 3 ISA Group, 201 North Union Street, Suite 300, Alexandria, VA 22314 USA; 4 Center for Evidence Synthesis in Health, Department of Health Services, Policy, and Practice, Brown University School of Public Health, 121 South Main Street, Box G-S121-8, Providence, RI 02903 USA; 5 Center for Evidence Synthesis in Health, Department of Health Services, Policy, and Practice, Brown University School of Public Health, 121 South Main Street, Box G-S121-8, Providence, RI 02903 USA; 6 Department of Epidemiology, Johns Hopkins Bloomberg School of Public Health, 615 North Wolfe Street, Baltimore, MD 21205 USA

**Keywords:** Systematic review, Outcomes, Meta-analysis, Core outcome sets, Loss of information, Clinical trials

## Abstract

**Background:**

There is broad recognition of the importance of evidence in informing clinical decisions. When information from all studies included in a systematic review (“review”) does not contribute to a meta-analysis, decision-makers can be frustrated. Our objectives were to use the field of eyes and vision as a case study and examine the extent to which authors of Cochrane reviews conducted meta-analyses for their review’s pre-specified main outcome domain and the reasons that some otherwise eligible studies were not incorporated into meta-analyses.

**Methods:**

We examined all completed systematic reviews published by Cochrane Eyes and Vision, as of August 11, 2017. We extracted information about each review’s outcomes and, using an algorithm, categorized one outcome as its “main” outcome. We calculated the percentage of included studies incorporated into meta-analyses for any outcome and for the main outcome. We examined reasons for non-inclusion of studies into the meta-analysis for the main outcome.

**Results:**

We identified 175 completed reviews, of which 125 reviews included two or more studies. Across these 125 reviews, the median proportions of studies incorporated into at least one meta-analysis for any outcome and for the main outcome were 74% (interquartile range [IQR] 0–100%) and 28% (IQR 0–71%), respectively. Fifty-one reviews (41%) could not conduct a meta-analysis for the main outcome, mostly because fewer than two included studies measured the outcome (21/51 reviews) or the specific measurements for the outcome were inconsistent (16/51 reviews).

**Conclusions:**

Outcome choice during systematic reviews can lead to few eligible studies included in meta-analyses. Core outcome sets and improved reporting of outcomes can help solve some of these problems.

## Background

There is broad recognition of the importance of evidence in determining clinical decision-making [1]. For evidence-based healthcare, decision-makers (e.g., patients, clinicians, guideline developers) increasingly rely on systematic reviews (“reviews”) [1]. Reviews identify primary studies, such as clinical trials and observational studies, that have addressed the research question of interest. This research question typically defines the population, interventions, and comparators; these defined aspects in turn help delineate the primary studies eligible for the review.

Reviews may or may not include quantitative syntheses of data across studies (“meta-analyses”). When appropriately conducted, meta-analyses provide decision-makers with summary estimates (e.g., relative risks) and accompanying estimates of uncertainty (e.g., 95% confidence intervals) that convey information about treatment effectiveness or safety succinctly [2]. Often, however, meta-analyses cannot be conducted because the studies address somewhat different clinical questions, assess different outcomes than the systematic reviewer (“reviewer”) had pre-specified, are methodologically heterogeneous, or are poorly-reported (e.g., inadequate information about results). In these circumstances, a study may be eligible for the review, but may not contribute to a meta-analysis [3]. When a review includes multiple studies, but these studies cannot be included in the meta-analysis, both doers (i.e., reviewers) and users of reviews (i.e., decision-makers) can be frustrated. Decision-makers want to know how treatments compare quantitatively; they may not be able to get reliable information about this when only some included studies contribute data to the meta-analysis or when no meta-analysis is possible [4].

Outcomes are measures or events used to assess the effectiveness and/or safety of clinical interventions [5]. A frequent reason for non-conduct of meta-analyses is that the studies assess different outcomes or assess the same outcomes, but do so differently. These scenarios can occur even among high-quality studies.

Although outcomes are fundamental to reviews of interventions, outcomes are typically not considered when determining the *eligibility* of a primary study in such reviews [6]. This is because outcomes inform meta-analyses, not whether the primary study is eligible for the review. Consistent with guidance in the Cochrane Handbook for Systematic Reviews of Interventions [6], we believe that studies that address the population, interventions, and comparators of interest should be included and cataloged in systematic reviews even if they do not report outcomes of interest. Outcome *choice* in a review is crucial because: (1) outcomes serve as yardsticks for basing conclusions about treatments; and (2) which outcomes are chosen and how they are defined can impact how many meta-analyses can be done and how many studies can be included in them [7–11].

Outcomes may be assessed differently in different studies because an “outcome” (a seemingly monolithic entity) actually comprises five elements: domain, e.g., visual acuity; specific measurement, e.g., Snellen chart; specific metric, e.g., ≥3 lines of vision lost; method of aggregation, e.g., proportion; and time-points, e.g., 6 months [9, 12]. Another example of the application of this five-element framework to clearly specify a particular data point of interest related to the outcome of “anxiety” is mean (method of aggregation) change (specific metric) in anxiety (domain) measured through the Hamilton Anxiety Rating Scale (specific measurement) from baseline to 1 year (time-point) [9, 12].

We previously demonstrated, through case studies in the fields of eyes and vision [11] and HIV/AIDS [10], that reviewers and clinical trialists addressing the same research question often examine different outcomes. In addition, inconsistency in outcome reporting across eligible studies prevents incorporation of all eligible studies into meta-analyses. For instance, a 2017 Cochrane systematic review comparing non-steroidal anti-inflammatory drugs (NSAIDs) with corticosteroids for inflammation after cataract surgery [13] included 48 trials, none of which reported data for the review’s pre-specified primary outcome, “proportion of patients with intraocular inflammation at 1 week after surgery.”

To document the extent and determinants of this problem, we embarked on the current case study in the field of eyes and vision. Our objectives were to examine the extent to which Cochrane reviews in eyes and vision conducted meta-analyses for the main outcome domain and the reasons why some otherwise eligible studies were not incorporated into meta-analyses.

## Methods

### Reviews examined

We examined all completed systematic reviews published by Cochrane Eyes and Vision in the Cochrane Database of Systematic Reviews as of August 11, 2017. We excluded reviews that were still in the protocol stage.

### Data extraction

We developed a data extraction form in the Systematic Review Data Repository (SRDR), an open-source platform for extracting and archiving data [14, 15]. Using a pilot-tested form, two individuals (from among SM, HK, BTS, and IJS) independently extracted data, resolving discrepancies through discussion. We extracted the following data: year published, population (i.e., eye function/region affected), and types of interventions and comparators. We extracted the numbers of primary, secondary, and other, i.e., non-primary and non-secondary, outcome domains. We also extracted the number of studies included in the review and in ≥1 meta-analysis for any, any primary, any secondary, and any other domain.

### “Main” outcome domains

We categorized one domain from each review as its “main” outcome domain (Table 1). For reviews that named only one primary outcome domain, we categorized it as the main outcome domain; for reviews that named more than one primary outcome domain (or named more than one secondary outcome domain), we categorized the primary outcome domain (or secondary outcome domain) with the highest number of included studies as the main outcome domain. For reviews that did not name any primary or secondary outcome domains, we categorized the “other”, i.e., nonprimary and non-secondary, outcome domain with the highest number of included studies as the main outcome domain.

**Table 1:** Algorithm for categorizing the “main” outcome domain for each systematic review

Scenario	If	Then	Number of systematic reviews (*N* = 175)
			n (%)
1	The review named only 1 primary outcome domain	we categorized that outcome domain as the main outcome domain.	131 (75)
2	The review named >1 primary outcome domain	we categorized the primary outcome domain with the highest number of included studies as the main outcome domain.	41 (23)
3	The review did not name any primary outcome domain, but named ≥1 secondary outcome domain	we categorized the secondary outcome domain with the highest number of included studies as the main outcome domain.	0 (0)
4	If the review did not name any primary or secondary outcome domains	we categorized the “other” (i.e., non-primary and non-secondary) outcome domain with the highest number of included studies as the main outcome domain.	3 (2)

Note

In scenarios 2, 3, and 4, if there were two or more possible outcome domains that had the same number of included studies (“Then” column), we categorized the first outcome listed in the Methods section as the main outcome domain

For each main outcome domain, we extracted the other four elements specified: specific measurement, specific metric, method of aggregation, and time-points. For the main outcome domain, we also extracted the numbers of studies that *reported measuring* it, *reported any data*, *reported any meta-analyzable data*, and were *incorporated into ≥ 1 meta-analysis*. We considered data for a given outcome from a given study to be “meta-analyzable” if the study reported adequate information so that it could be incorporated into a meta-analysis. For categorical outcomes, meta-analyzable meant that either of these conditions were met: (1) total number of participants and number of participants with the outcome were reported for each study arm; and (2) the between-group treatment effect (e.g., relative risk) and an uncertainty estimate (e.g., 95% confidence interval) were reported. For continuous and time-to-event outcomes, meta-analyzable meant that either of these conditions were met: (1) mean and uncertainty estimates were reported for each study arm; and (2) the between-group treatment effect (e.g., mean difference) and an uncertainty estimate were reported.

## Results

### Reviews examined

We identified 175 completed systematic reviews published by Cochrane Eyes and Vision in the Cochrane Database of Systematic Reviews (Table 2). The reviews were published between January 1, 2005 and August 11, 2017 (median = 2014). The most common populations were patients with retinal/choroidal disease (35 reviews; 20%) and visual impairment/low vision (33 reviews; 19%). The most common types of interventions/comparators were drugs (74 reviews; 42%) and surgeries (67 reviews; 38%).

**Table 2:** Characteristics of systematic reviews examined

Characteristic	Number of systematic reviews (*N* = 175)
	n (%)
*Year published*	
2003–2005	3 (2)
2006–2008	12 (7)
2009–2011	15 (9)
2012–2014	68 (39)
2015–2017	77 (44)
*Population (function/region of eye) addressed*	
Retinal/choroidal disease	35 (20)
Visual impairment/low vision	33 (19)
Optic nerve, including glaucoma	32 (18)
Ocular surface	31 (18)
Lens	18 (10)
Ocular vasculature	5 (3)
Other	21 (12)
*Interventions and comparators examined*^*a*^	
Drug	74 (42)
Surgery	67 (38)
Other procedure	31 (18)
Device	15 (9)
Supplements	6 (3)
Screening/testing	5 (3)
Other intervention	26 (15)
*Number of outcome domains examined*	
Median	6
Interquartile range	5 to 8
Range	1 to 19
*Number of primary outcome domains examined*	
Median	1
Interquartile range	1 to 1
Range	0 to 5
*Number of secondary outcome domain examined*	
Median	4
Interquartile range	3 to 6
Range	0 to 12
*Number of other outcome domains examined*	
Median	1
Interquartile range	0 to 2
Range	0 to 6
*Number of studies included*	
Median	3
Interquartile range	1 to 9
Range	0 to 137

^a^More than one category could apply

### Incorporation of studies into meta-analyses for any outcome domain

The 175 included reviews examined a median of 6 total outcome domains, including a median of 1 primary outcome domain, 4 secondary outcome domains, and 1 other outcome domain.

The 175 reviews included a median of 3 studies (IQR 1–9); 125 reviews (71%) included ≥2 studies. For these 125 reviews, Fig. 2 plots the percentage of studies incorporated into a meta-analysis for any outcome domain (blue line) and for the main outcome domain (red bars). Among these reviews, 44/125 reviews (35%) incorporated every included study into ≥1 meta-analysis (for any outcome domain). Conversely, 33/125 reviews (26%) did not incorporate any study into any meta-analysis for any outcome, i.e., they did not conduct any meta-analysis. The remaining 48/125 reviews (38%) incorporated only a subset of their studies into ≥1 meta-analysis. These 48 reviews included a median of 12.5 studies (IQR 6–22), and the meta-analyses in these reviews incorporated a median of 6.5 studies (IQR 4–13).

Among the 125 reviews that *could have conducted* a meta-analysis, i.e., those including ≥2 studies, the median proportion of studies incorporated into ≥1 meta-analysis for any outcome was 74% (IQR 0–100%). Among the 92 reviews that *conducted* a meta-analysis, the median proportion of studies incorporated into ≥1 meta-analysis for any outcome was 93% (IQR 64–100%).

### Characteristics of main outcome domains

Almost all reviews (172/175 reviews; 98%) named ≥1 primary outcome domain (Table 1). Three in four reviews (131/175 reviews; 75%) each named exactly one primary outcome domain, which we categorized as their main outcome domain. The most frequent main outcome domains across the 175 reviews were visual acuity (31%) and intraocular pressure (6%) (Table 3). Thirty-eight outcome domains were main outcome domains in just one review each. The main outcome was categorical in 70% and continuous in 29% of reviews. Most main outcome domains (98%) were efficacy outcomes, i.e., not safety outcomes.

**Table 3:** Characteristics of main outcome domains in all 175 systematic reviews examined

Characteristic	Number of systematic reviews (*N* = 175) n (%)
*Main outcome domain*	
Visual acuity	55 (31)
Intraocular pressure	11 (6)
Visual field	7 (4)
Visual impairment/vision loss	5 (3)
Success of surgery/procedure	5 (3)
Failure of trabeculectomy	4 (2)
Progression of age-related macular degeneration	3 (2)
Reading speed	3 (2)
Ocular symptoms (unspecified)	3 (2)
Symptoms of dry eye	3 (2)
Vision-related quality of life	3 (2)
Resolution of infection	3 (2)
Active trachoma	3 (2)
Healing of keratitis	3 (2)
Other	64 (37)
*Type of main outcome domain*	
Categorical	122 (70)
Continuous	50 (29)
Other (i.e., time-to-event)	2 (1)
Not reported	1 (0)
*Goal of main outcome domain*	
Efficacy	172 (98)
Safety	3 (2)

### Incorporation of studies into meta-analyses for the main outcome domain

Among the 125 reviews including ≥2 studies, only 18 reviews (14%) incorporated all their studies into a meta-analysis for the main outcome domain. Conversely, 51/125 reviews (41%) did not incorporate any study into the meta-analysis for the main outcome domain, i.e., they did not conduct any meta-analysis for the main outcome domain. The remaining 56/125 reviews (45%) incorporated only a subset of their studies into the meta-analysis for the main outcome domain. These 56 reviews included a median of 12 studies each, and the meta-analyses for the main outcome domain in these reviews incorporated a median of 4 studies each.

Among the 125 reviews that *could have conducted* a meta-analysis, i.e., those including ≥2 studies, the median proportion of studies incorporated into ≥1 meta-analysis for the main outcome domain was 28% (IQR 0–71%). Among the 74 reviews that *conducted* meta-analyses for the main outcome domain, the median proportion of studies incorporated was 67% (IQR 39–91%).

### Meta-analysis conduct for the main outcome domain

**Fig. 1:**
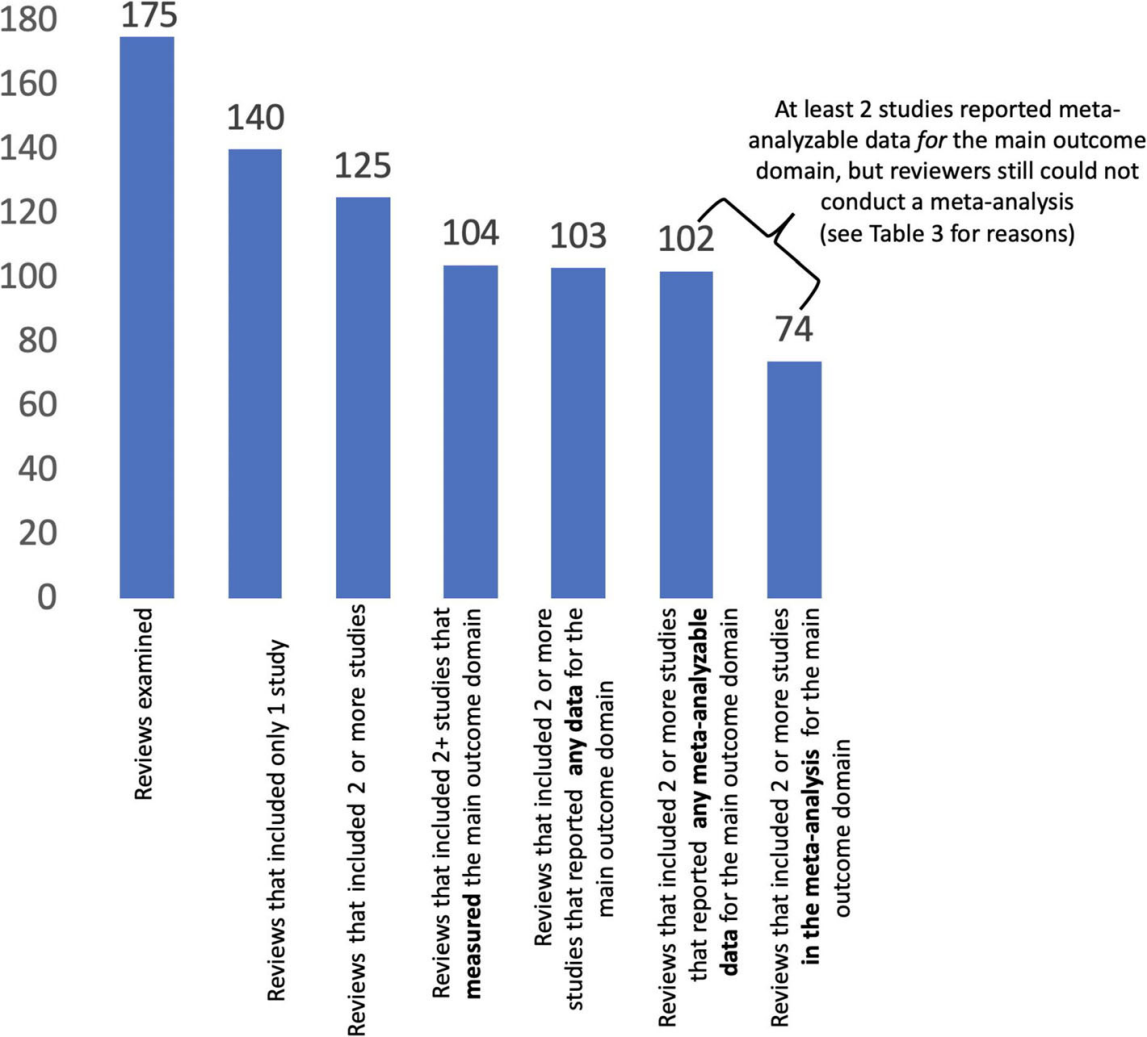
Conduct of meta-analyses for the main outcome domain

Figure 1 illustrates a cascading effect of loss of information as regards the main outcome domain in the 175 reviews. Thirty-five reviews (20%) included no studies, i.e., were empty reviews, and 15 (9%) included one study each (Fig. 1). Of the 125 reviews including ≥2 studies, i.e., those in which a meta-analysis could theoretically be done for the main outcome if ≥2 studies reported meta-analyzable data, only 74 reviews (59%) conducted a meta-analysis for the main outcome.

### Reasons for non-conduct of meta-analyses for the main outcome domain

Among the 125 reviews including ≥2 studies, 51 reviews (41%) did not conduct a meta-analysis for the main outcome domain. For 21/51 reviews (41%), fewer than two studies measured the review’s main outcome (Table 4). When ≥2 studies reported meta-analyzable data, there were numerous reasons why reviewers did not conduct a meta-analysis, most frequently due to inconsistency in outcome elements among the included studies. Specifically, data could not be meta-analyzed because the specific measurements used (16/51 reviews; 31%) and time-points examined (9/51 reviews; 18%) were inconsistent among studies.

**Table 4:** Reasons for non-conduct of a meta-analysis for the systematic review’s main outcome even when ≥2 studies were included in the systematic review (*N* = 51 of 125 reviews that included ≥2 studies)

Reason	Number of systematic reviews (*N* = 51)	
	n (%)	
When meta-analyzable data^1^ for the review’s main outcome domain were NOT REPORTED by ≥2 studies (*n* = 23 reviews)		
< 2 studies *measured* the review’s main outcome	21	(41)
< 2 studies *reported any data for* the review’s main outcome	1	(2)
< 2 studies *reported any meta-analyzable data*^1^*for the review’s* main outcome	1	(2)
When meta-analyzable data^1^ for the review’s main outcome domain were REPORTED by ≥2 studies (*n* = 28 reviews)^2^		
Reasons related to inconsistencies in outcome elements		
Studies used *inconsistent specific measurements*	16	(31)
Studies used *inconsistent specific metrics*	0	(0)
Studies used *inconsistent methods of aggregation*	0	(0)
Studies reported data at *inconsistent time-points*	9	(18)
Reasons related to heterogeneity		
Studies were *clinically* heterogeneous	7	(14)
Studies were *methodologically* heterogeneous	2	(4)
Studies were *statistically* heterogeneous	0	(0)

^1^For categorical outcomes, we considered data to be meta-analyzable if either of the following scenarios were met [1]: total number of participants and number of participants with the outcome of interest were reported for each study arm; and [2] the between-group treatment effect (e.g., relative risk, odds ratio) and an estimate of uncertainty (e.g., 95% confidence interval) were reported. For continuous and time-to-event outcomes, we considered data to be meta-analyzable if either of the following scenarios were met [1]: mean and estimate of uncertainty (e.g., standard deviation) were reported for each study arm; and [2] the between-group treatment effect (e.g., mean difference) and an estimate of uncertainty (e.g., 95% confidence interval) were reported

^2^More than one reason could apply

**Fig. 2:**
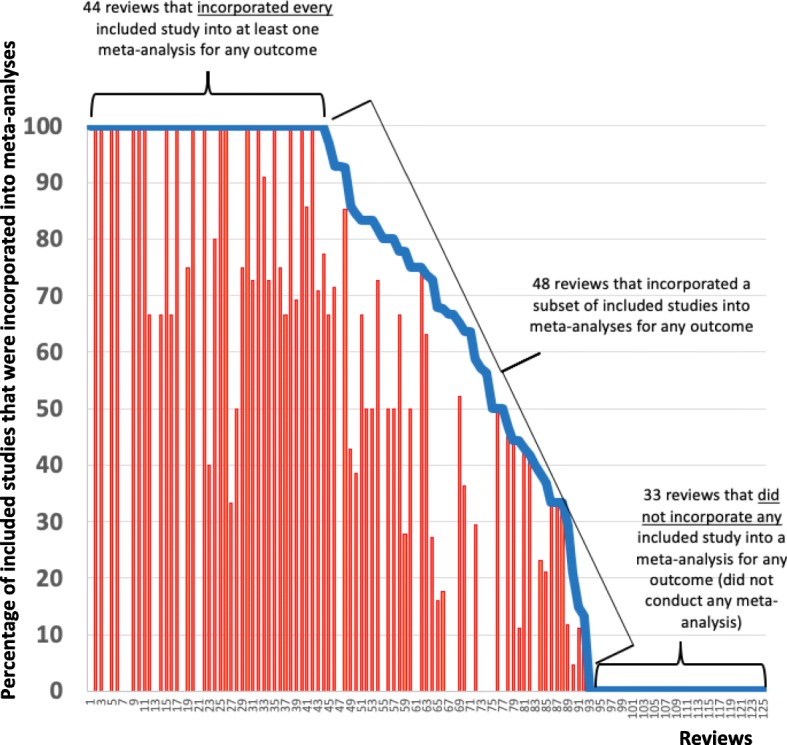
Percentage of studies included in the review that were incorporated into a meta-analysis for any outcome (blue line) and for the review’s main outcome (red bars) Notes: This Figure excludes the 50 systematic reviews in whom a meta-analysis was not possible: 35 systematic reviews that each included 0 studies (i.e., “empty reviews”) and 15 systematic reviews included that each included only 1 study. When the blue line is non-0 but the red bars are 0, it implies that the systematic review did not conduct a meta-analysis for the main outcome, but did so for ≥1 of the remaining outcomes

Figure 2 demonstrates that the loss of information for the main outcome domain (red bars) was similar in pattern to the loss of information when considering any outcome domain (blue line).

## Discussion

Through a case study of all Cochrane reviews in the field of eyes and vision, the current work demonstrates three major areas that need improvement.

First, primary studies addressing similar research questions should align their outcomes better. Studies often could not be incorporated into meta-analyses because the outcomes were not aligned, either because the domains or ≥1 of the other four outcome elements did not overlap. Among the reviews including ≥2 studies, only 59 and 74% could conduct a meta-analysis for the main outcome and for any outcome, respectively. In other words, even when reviews included ≥2 studies, 41 and 26% of reviews missed opportunities to conduct a meta-analysis to succinctly convey information regarding the main outcome and any outcome, respectively.

Second, reviews and primary studies should align their outcomes better. When looking at reviews that could have conducted a meta-analysis, i.e., those including ≥2 studies, the median percentages of included studies incorporated into the meta-analysis for the main outcome and for any outcome were 28 and 74%, respectively. This suggests that, approximately 7 in 10 studies that reviewers include are not incorporated into the meta-analysis for the *main* outcome, and 1 in 4 studies are not incorporated into the meta-analysis for *any* outcome. In previous work, we demonstrated poor overlap between outcomes in clinical trials and reviews, and possible differences in the types of outcomes they examine [10, 11]. For HIV/AIDS, we demonstrated that reviewers examined more long-term clinical outcomes and patient-centered outcomes than did clinical trialists. Such differences may arise because: (1) reviews may more directly inform clinical practice guidelines, and (2) reviewers may be less affected by common constraints faced by clinical trialists, e.g., costs and sample size [10].

Our findings beg the question of who should prioritize outcomes for measurement and reporting in research. It has aptly been stated that achieving consensus in outcome use across research “cannot be left to serendipity.” [16] One deliberate and fundamental aspect of the solution to the problem of outcome inconsistency is the development of “core outcome sets.” A core outcome set is a minimum set of outcomes that should be measured and reported in all clinical trials addressing a given condition [17]. Core outcome sets are increasingly common in various health fields; a 2018 systematic review identified 307 core outcomes sets [18]. However, outcome inconsistency remains widespread; 40% of recent (2019) published Cochrane reviews explicitly noted this problem [19].

We [10, 11] and others [20] have argued that, as stakeholders in a given field, systematic reviewers should both participate in the development of and adopt core outcome sets for that field. By broadening the participation in outcome prioritization efforts, this could potentially help ensure that the outcomes that are measured and reported in research are widely relevant and important. Two aspects of core outcome sets are worthy of clarification. First, core outcome sets do not stifle innovation; they are simply meant to represent a minimum set of outcomes that should be reported. Once a core outcome set exists for a given topic, clinical trialists working in that topic area should explicitly specify the intention to measure and report the outcomes in the set. Second, core outcome sets are not static; they can and should be updated as the field advances and new knowledge emerges.

The third major area in need of improvement that our study demonstrates is the reporting of outcomes in primary studies. Results data from primary studies were often not meta-analyzable even when outcomes might have been aligned. In addition, outcome domains were frequently not reported in primary studies or ≥1 of the outcome elements were frequently missing or inadequately reported (e.g., “worsening of disease” without clarification of how “worsening” was defined). It is possible that the studies measured these outcomes, but did not report measuring them or reported them inadequately. If such selective reporting, either non-reporting or inadequate reporting, of outcomes in the included studies occurred as a function of the direction of the outcome’s results, it would be suggestive of *outcome reporting bias* [21]. In this case study, we relied on the reviewers’ reporting of the extent to which the primary studies reported the outcomes. Because we did not examine the reports of the primary studies (or their protocols), we are unable to comment definitively on whether non-reporting of the outcomes indicates outcome reporting bias. However, outcome reporting bias in primary studies has been documented to be a widespread problem across reviews [22–26], and, as such, is a likely explanation for some outcomes not being reported.

### Implications

For the evidence-based medicine paradigm to work, decision-makers must be able to rely on systematic reviews, which in turn rely on the results of primary studies. For results of primary studies to be actionable, there (1) needs to be alignment in outcomes considered important to both primary study researchers and reviewers, and (2) those outcomes need to be reported completely. Important discussions need to be had regarding who should choose outcomes for the field and how such choices should be made. We, in conjunction with others, suggest that these discussions should include, at the least, clinicians, patients, clinical trialists, systematic reviewers, regulators, and other decision-makers [27].

We have demonstrated that the choice of outcomes for systematic reviews may have led to loss of information through non-incorporation of results from included studies into meta-analyses. The most substantial drops in the percentage of reviews conducting meta-analyses for the main outcome domain appeared to be due to inadequate numbers of studies reporting the outcome and, when there were adequate numbers of studies for a meta-analysis (i.e., ≥2 studies), differences in the specific measurements and time-points used.

Our findings also demonstrate that even when focusing on reviews that conducted meta-analyses for their main outcome domain, only about 2 in 3 studies were incorporated into those meta-analyses. As such, non-incorporation of included studies into meta-analyses represents two main problems. First, it represents missed opportunities for using research to inform decision-making through evidence synthesis. This contributes considerably towards research waste [28–30]. Second, non-incorporation of included studies into meta-analyses represents a failed obligation on the part of the researchers (both trialists and reviewers) [31]. As a community of researchers, both parties have a solemn obligation to research participants to ensure that their participation will lead to a useful contribution to science; failing to agree upon outcomes that should be collected and adequately reported likely violates this obligation.

### Other solutions

Core outcome sets are integral to solving the problems this study illustrates. Other parts of the solution are worth discussing. We agree with existing recommendations against studies being excluded from systematic reviews solely on the basis of the lack of relevant outcome data [3]. Thankfully, such recommendations have been associated with a reduction in the number of reviews excluding studies solely on the basis of outcome data [32]. As the current study demonstrates, the review team’s choice of outcomes may not align with that of the primary studies. This may be particularly true for eyes and vision, a field with few core outcome sets [4, 18]. We also encourage reviewers to report an outcome matrix [23, 24], a transparent and simple way to indicate all fully-reported, partially-reported, or non-reported outcomes in each included study.

### Large numbers of empty reviews and reviews including only one study

Twenty-percent of the reviews we examined were empty and 9% included only one study each. While such reviews are useful in driving primary research, the possible reasons for the paucity of studies in them are worth exploring. One possibility is that these represent topics that primary researchers have not yet studied. Another is that only observational studies addressing these topics may exist; Cochrane reviews typically include only randomized trials. It also is possible that these topics reflect the priorities of Cochrane Eyes and Vision and the authors of these reviews, rather than of the field at-large.

### Limitations

Our study has certain limitations. First, we focused on Cochrane reviews within one field. Loss of information due to the choice of review outcomes could be a bigger, similar, or smaller problem in non-Cochrane systematic reviews in eyes and vision or systematic reviews in other fields. Second, we analyzed in-depth the extent of incorporation of included studies into meta-analyses only for the main outcome domain. Meta-analyses of other primary, secondary, and other outcome domains may have incorporated higher percentages of included studies. However, Fig. 2 suggests that this is likely not the case. It is possible that our algorithm for categorizing the “main” outcome for each review could have impacted our findings. But, in reviews where more than one outcome domain could have served as the main outcome, we categorized as the main outcome the outcome that the highest number of included studies had reported. Our results thus represent the best-case scenario. Third, most outcome domains (98%) were efficacy outcomes. Selective outcome reporting has also been reported to be a problem for safety outcomes [33]. Fourth, we relied on the reviews to determine whether or not each included study did the following for the main outcome domain: reported measuring it, reported any results for it, and reported meta-analyzable data for it. Related to this, we did not examine the appropriateness or feasibility of the reviewers’ being able to conduct meta-analyses when the included studies reported data in a format different from what the reviewers were interested. As such, our results document what was actually done in the reviews.

## Conclusions

This case study of all Cochrane systematic reviews addressing an entire field (eyes and vision) demonstrates that only 59 and 74% of the reviews including ≥2 studies could conduct a meta-analysis for the main outcome and for any outcome, respectively. In evidence-based healthcare, such loss of information represents missed opportunities and a failed obligation by researchers to research participants to ensure that their participation will lead to a useful contribution to science. Core outcome sets and improved outcome reporting can help solve some of these problems.

## Data Availability

The datasets used and/or analysed during the current study are available from the corresponding author on reasonable request.

## Abbreviations

IQR: Interquartile range
NSAID: Non-steroidal anti-inflammatory drug
SRDR: Systematic Review Data Repository
